# Design of an expression system to enhance MBP-mediated crystallization

**DOI:** 10.1038/srep40991

**Published:** 2017-01-23

**Authors:** Tengchuan Jin, Watchalee Chuenchor, Jiansheng Jiang, Jinbo Cheng, Yajuan Li, Kang Fang, Mo Huang, Patrick Smith, Tsan Sam Xiao

**Affiliations:** 1 Laboratory of Structural Immunology, CAS Key Laboratory of Innate Immunity and Chronic Diseases, CAS Center for Excellence in Molecular Cell Sciences, School of Life Sciences and Medical Center, University of Science and Technology of China, Hefei 230027 China; 2 Laboratory of Immunology, National Institute of Allergy and Infectious Diseases, National Institutes of Health, Bethesda, MD 20892 USA; 3 Department of Pathology, Case Western Reserve University, Cleveland, OH 44106 USA

## Abstract

Crystallization chaperones have been used to facilitate the crystallization of challenging proteins. Even though the maltose-binding protein (MBP) is one of the most commonly used crystallization chaperones, the design of optimal expression constructs for crystallization of MBP fusion proteins remains a challenge. To increase the success rate of MBP-facilitated crystallization, a series of expression vectors have been designed with either a short flexible linker or a set of rigid helical linkers. Seven death domain superfamily members were tested for crystallization with this set of vectors, six of which had never been crystallized before. All of the seven targets were crystallized, and their structures were determined using at least one of the vectors. Our successful crystallization of all of the targets demonstrates the validity of our approach and expands the arsenal of the crystallization chaperone toolkit, which may be applicable to crystallization of other difficult protein targets, as well as to other crystallization chaperones.

Recombinant expression of fusion proteins containing a target protein and an unrelated expression tags is a common strategy to obtain samples for medical and research applications^1^. Depending on their function and properties, these tags can be divided into several categories including purification tags, stability tags, and function tags. Many proteins have been tested as expression tags including the glutathione S-transferase (GST), small ubiquitin-like modifier (SUMO), thioredoxin (TRX), disulfide oxidoreductase (DsbA), N utilization substance protein A (NusA), B1 domain of protein G (GB1), oligopeptide derived from pancreatic ribonuclease A (S-tag), and maltose-binding protein (MBP)^2^.

MBP is one of the most commonly used protein expression tags due to its exceptional performance in enhancing the solubility of the target proteins^3^. It is the periplasmic portion of the ATP-binding cassette (ABC) maltose/maltodextrin transporter found in many bacterial species^4^. MBP has a large protein-protein interaction surface on the same side of the maltose ligand-binding pocket, through which it binds to the transmembrane domain of the ABC transporter. It is commonly used as an affinity tag for purification, which takes advantage of its ability to bind amylose. While the mechanism of the solubility-enhancing property of MBP is still not completely understood, its large size, intrinsic solubility, abundance of flexible loops, and high percentage of exposed hydrophilic residues may be contributing factors. Recent studies indicate that MBP does not have chaperone activity originally thought to facilitate the folding of the fused proteins^5^.

Obtaining diffraction-quality crystals remains the rate-limiting step in solving a novel structure using X-ray crystallography, despite the recent advancements in the areas of molecular cloning, protein production and purification, crystallization screening, data collection using synchrotron radiation, and structure determination programs. Many tools have been developed and employed in the structural biology community to enhance the crystallizability of proteins, including *in situ* proteolysis^6^,^7^,^8^, lysine methylation^9^,^10^,^11^, surface entropy reduction^12^,^13^,^14^, metal mediated crystallization^15^, polymer-driven crystallization^16^, site-specific mutagenesis^17^, and chaperone-aided crystallization (also see recent reviews refs 18,19). In particular, many high-affinity binding partner molecules have been used to facilitate the crystallization of challenging protein targets such as the G protein-coupled receptors (GPCRs). These high-affinity binders also include antibody derivatives, such as Fab and scFv, and other alternative scaffolds that have a high affinity for the target proteins^20^. In addition, covalently linked protein tags, either at the termini or within a flexible loop of the target protein, have also been shown to promote the formation of crystallization contacts. Many protein tags have been tested and successfully used as crystallization chaperones. These protein tags include the T4 lysozyme^21^,^22^, apocytochrome b562RIL^23^,^24^,^25^, GST^26^, TRX^27^, green fluorescent protein (GFP)^28^,^29^, SUMO (pdb: 3V7O and 4G50), and MBP (Table S1).

The first report using MBP to facilitate crystallization of unrelated protein/peptide dates back to 1998, when a 26 amino acid-long peptide from a dominant B-cell epitope sequence of a hepatitis B surface antigen was crystallized in the form of an MBP fusion^30^. The first protein that was crystallized with MBP was the human T cell leukemia virus type 1 GP21 in 1998^31^,^32^. A few important proteins, otherwise recalcitrant to crystallization, have been fused to MBP to obtain their first structures, such as the GPCR extracellular domains^33^. Moreover, MBP fusion technique has been used in the structure determination of several protein: protein complexes^34^,^35^,^36^. The synergistic effects of combining fixed-arm linkers and surface entropy reduction mutations in MBP were demonstrated recently^13^. The advantage of using a short linker instead of a long one was demonstrated by several studies^13^,^30^. To explore the method of fusion tag-mediated crystallization, the biggest challenge is still the design of such chimeric expression constructs to maximize the crystallizability of the targets^18^. Waugh stated in a recent review on crystal structures of MBP fusion proteins that a short helical linker (i.e., NAAA) was a frequently used linker^37^. Despite that using MBP tag as an expression and crystallization platform is increasingly appreciated^38^,^39^, the optimal sequence, length, and structure of the linker between MBP and the target proteins have not been fully investigated, which limits the application of this crystallization strategy.

The death domain superfamily is a structural motif found in many proteins that are involved in physiological, as well as pathological processes. It can be divided into four different subfamilies: caspase recruitment domain (CARD), death domain (DD), death effector domain (DED), and the pyrin domain (PYD)^40^. These death fold domains often participate in protein-protein interactions leading to the formation of large, oligomeric signaling complexes such as the apoptosome and inflammasome^40^. Structural information is available for only about a third of the total 99 death domain superfamily members in the human genome, and seven death-fold protein complex structures have been determined through X-ray crystallography, including the PIDDosome^41^, FAS/FADD-DISC^42^,^43^, MyDDosome^44^, and apoptosome^45^. Structural characterization of these death domains and their complexes is often challenging because of their low stability and tendency to self-interact and aggregate. Inspired by the successful examples of using MBP as a crystallization chaperone, we designed an expression vector (V28E) coding an *E. coli* MBP protein^13^,^46^ followed by a very short linker with amino acid sequence of valine and aspartate encoded by the Sal I restriction enzyme site (GTCGAC). This vector has limited success in expression and crystallization of several fusion proteins for PYD and CARD domains^47^,^48^,^49^. Here we sought to systematically explore the usage of linkers of various lengths and test their effects on crystallization of death domain superfamily members. We hypothesize that optimization of the linker regions between MBP and the target protein will increase the likelihood of obtaining crystals. To this end, we designed a set of seven expression vectors harboring different helical linker sequences, and selected seven death domains as targets, six of which have never been crystallized before. The design of the linker sequences takes into consideration the rigidity of the helical structure, the various spacing and relative orientations of MBP and target proteins connected by different helical linkers, and the minimal structural perturbation by a helical linker that connects the C-terminal helix from MBP with the N-terminal helix from a death domain superfamily member. Our successful crystallization of all of the targets demonstrates the validity of our approach and expands the arsenal of the crystallization chaperone toolkit.

## Methods

### Data base mining

A BLAST search using the *E. coli* MBP sequence was performed with the Protein Data Bank (www.rcsb.org) to collect information on all deposited structures containing an MBP tag. All of the hits were manually inspected for information of the target proteins such as size and isoelectric point (pI).

### Design of the MBP expression vectors

The coding sequence for an *E. coli* MBP protein mutant “E” (D82A/K83A/E172A/N173A/K239A)^13^,^46^ was amplified by PCR and ligated into a T7 promoter-based bacterial protein expression vector pET30a (Novagen) using restriction enzyme sites Nde I and Xho I (sequence is shown in Table S2). A Sal I site was introduced following the MBP residue A369. This vector was designated “V28E” that features a short linker of residues VD encoded by the Sal I site. Building on vector V28E, vectors V28E1-E6 were designed to harbor helical links with one residue increment (Table 1). Gene of interest can be ligated into this set of vectors using the same restriction enzymes Not I and Xho I, with the Not I site encoding residues AAA that adopts helical structures. A hexa-histidine tag sequence following the Xho I site was retained from the pET30a backbone to facilitate the purification of the fusion protein through immobilized metal affinity chromatography (IMAC).

### Protein expression, purification and crystallization

Expression and purification of the MBP-tagged proteins have been described previously^47^,^48^. Briefly, transformed BL21 (DE3) Codon Plus RIPL cells (Stratagene, Santa Clara, CA) were grown at 37 °C and protein expression was induced at 18 °C for at least two hours with 0.3 mM IPTG. Cells were lysed by sonication and the recombinant protein was purified by IMAC columns followed by size-exclusion chromatography. The purified protein was concentrated to 20–50 mg/ml in the presence of 10 mM maltose before setting up crystallization screening.

Coding sequences for seven death domain superfamily members (hNLRP1-CARD, hAIM2-PYD, hCARD8-CARD, zIGBP1-CARD, hNLRP1-PYD, hNLRP12-PYD, and hMNDA-PYD) were ligated into each of the V28E and V28E1-E6 vectors. Several domain boundaries were tested for each of the targets, which resulted in more than 50 expression constructs (Table S2). Crystallization of these fusion proteins was screened using the Mosquito crystallization robot (TTP Labtech, United Kingdom). For a particular target protein, once the structure was determined using one of the constructs, further exploration of crystallization for the same target was terminated. It is therefore possible that some additional crystallization conditions for the target might be missed.

### X-ray diffraction data collection and structure determination

X-ray diffraction data were collected at GM/CA beamline at the Advanced Photon Source, Argonne National Laboratory (ANL) (Table S3). Data were processed with the HKL2000 program suite^49^ and XDS^50^. Structures were solved using molecular replacement program Phaser^51^ from the CCP4 program suite^52^ and an *E. coli* MBP structure (3DM0) as the search model. The structures were determined through alternative manual model building with Coot^53^ and refinement with Phenix^54^. The crystal structures were validated by the Molprobity server^55^. Figures were prepared using PyMol (The PyMOL Molecular Graphics System, Version 1.5.0.4 Schrödinger, LLC.).

## Results

### A survey of using MBP as a crystallization chaperone

**Figure 1:**
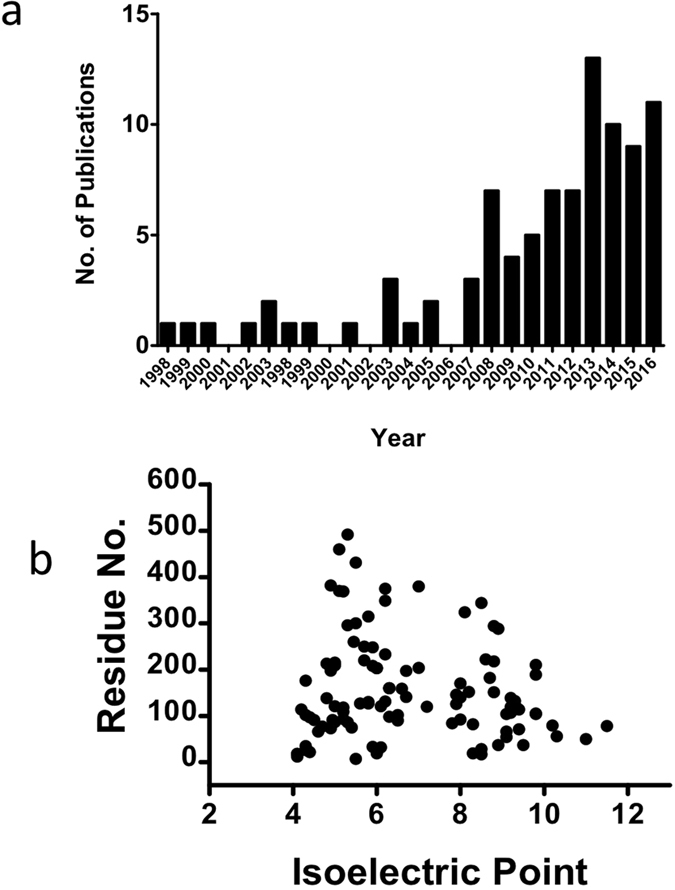
MBP is the most successful crystallization chaperone. (**a**) Number of publications reporting MBP fusion protein structures in recent years. (**b**) Target size distribution and isoelectric point of MBP fusion protein structures. The residue numbers of target proteins were manually verified from the deposited fusion protein structures. Linker and C-terminal artificial sequences such as cloning site and His-tag were not counted.

A survey of the Protein Data Bank (www.rcsb.org) showed that many groups have successfully adopted the MBP fusion strategy in the crystallization of their proteins of interest (Table S1). To date, there are over 50 primary research articles reporting the structures of MBP-tagged fusion proteins and there is still a clear upward trend (Fig. 1a). There are over 100 deposited MBP fusion protein structures in the PDB by the middle of 2016, making MBP one of the most utilized crystallization chaperone (also see Waugh’s recent review ref. 37). The MBP-tagged targets vary in their overall folds, function, and origins (Table S1). The size of the target proteins falls in the range of 12 residues to 431 residues, with the median of 105 residues (Fig. 1b). The isoelectronic points of target proteins are distributed between 4.0 and 11.0, with a medium value of 6.6 (Fig. 1b).

During our survey of the linker sequences between MBP and the target proteins, we noticed that most linkers are short. This suggests that the relative orientations of MBP and the target proteins may be restricted despite the largely random coil structures of the linker. In agreement with a recent review ref. 37, we have not been able to find consensus linker sequence in these structures, although a linker with the sequence AAA appears to be common.

### Crystallization of fusion proteins with a short VD linker

**Figure 2:**
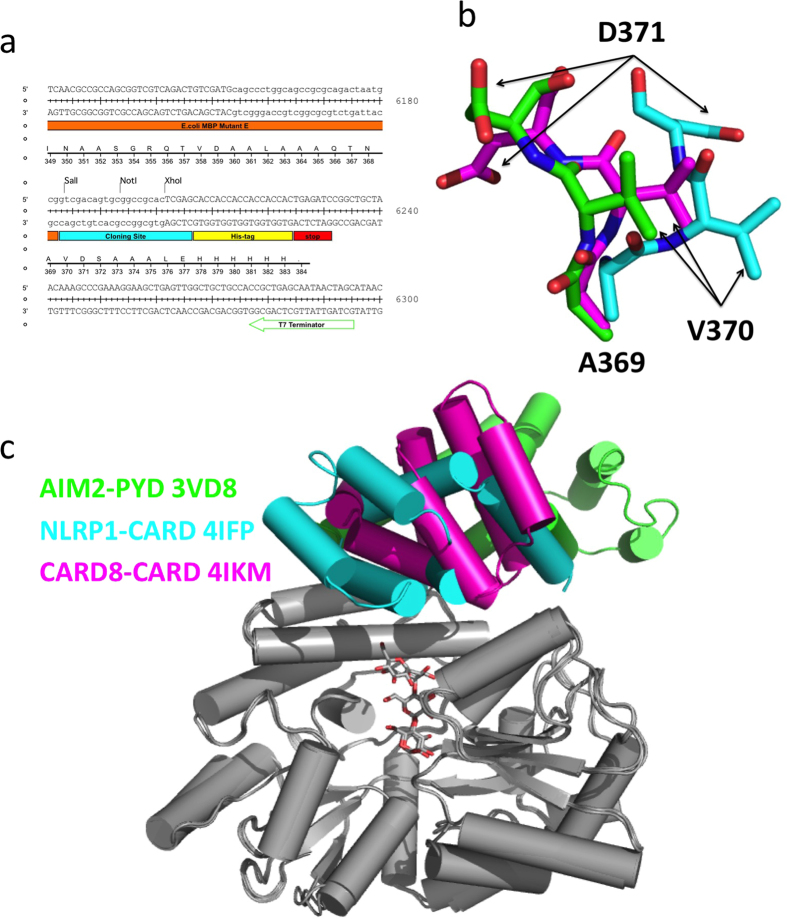
Crystallization of MBP fusion proteins with a short VD linker. (**a**) The cloning site of the V28E expression vector. Coding sequences for genes of interest can be ligated into the V28E vector using restriction enzyme sites of Sal I and Not I. The other six vectors V28E1-E6 share the overall feature as V28E but use the Not I and Xho I sites for insert ligation. (**c**) Superposition of the MBP tags in the three fusion protein structures. The three target proteins show different conformation relative to the MBP tag due to the flexibility of VD linker. (**b**) Different conformations of the linker loops from the three structures in (**c**).

**Figure 3:**
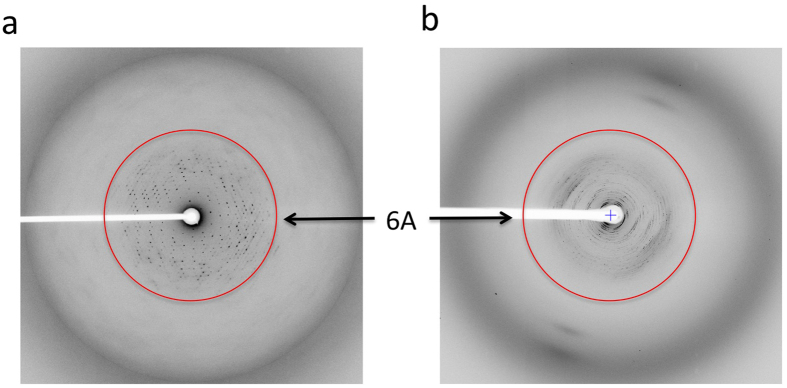
Structure of the zebrafish IGBP1-CARD crystallized using V28E5. (**a**) X-ray diffraction image of zebrafish IGBP1-CARD as MBP-fusion protein expressed with the V28E vector with VD linker. (**b**) X-ray diffraction image of human NLRP1-PYD as an MBP-fusion protein expressed with the V28E vector containing a VD linker.

In our study of the death domain superfamily members, obtaining highly purified samples for crystallization has been challenging, as documented for many of these protein-protein interaction domains^56^. Our initial design of the V28E vector (Fig. 2a) was based on an *E. coli* MBP protein mutant harboring five mutations that reportedly enhance its crystallizability^13^,^46^. The short linker of VD residues followed by the first helical residues of the target proteins may help enhance crystallization. Even though this strategy has resulted in success for some of our targets such as the NLRP1 CARD^47^, and CARD8 CARD^48^, and the AIM2 PYD domain^47^ (Fig. 2b,c), it has failed for other targets such as the zebrafish IGBP1-CARD and human NLRP1-PYD (sequences are listed in Table S2). After extensive testing of the types and concentrations of different commonly used cryo-protectant solutions, we failed to collect useful X-ray diffraction data suitable for structural determination despite the fact that sizable crystals were readily obtained (Fig. 3a,b). The poor diffraction quality of these MBP fusion proteins may be related to the VD linker that introduces internal flexibility of the fusion proteins and micro-scale heterogeneity of the crystals. Indeed, low resolution diffraction is often observed for crystals of large proteins or protein complexes due to structural complexity and internal flexibility^50^.

As expected, the VD linker region forms a loop bridging the MBP and target proteins in the three solved structures above (Fig. 2b,c). However, conformations of the linker loops among the three structures are drastically different, suggesting that the flexible VD linker may not be optimal for crystallization of some targets such as the IGBP1-CARD and NLRP1-PYD. In addition, target proteins, even those of similar size and fold, may significantly influence the overall structure of the fusion proteins.

### Crystallization of fusion proteins containing a series of helical linkers

**Figure 4:**
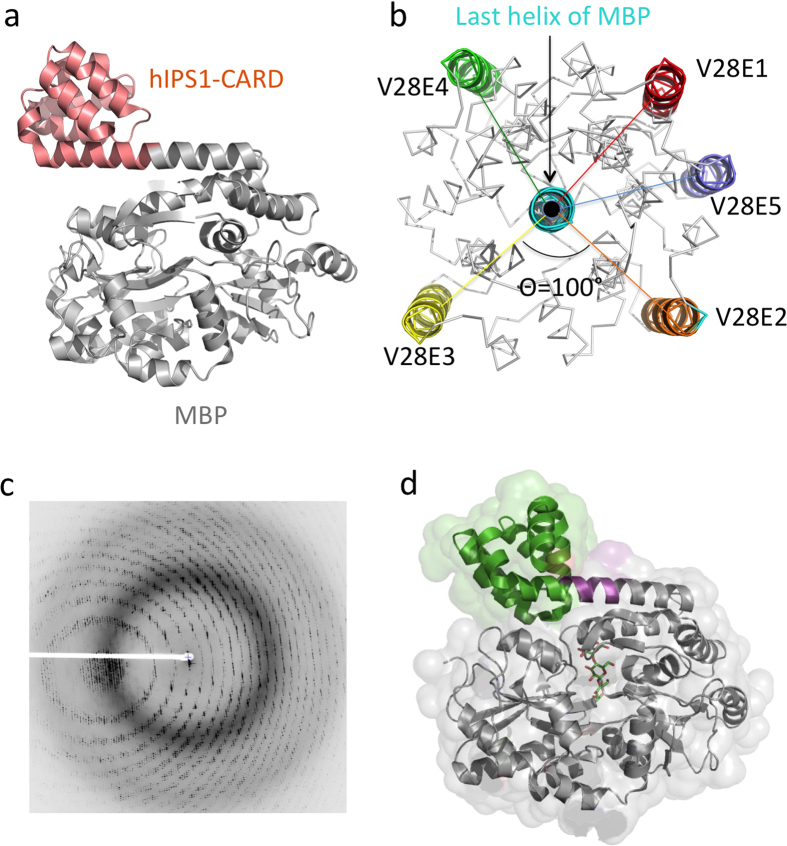
Design of helical linkers for MBP fusion proteins. (**a**) Cartoon representation of MBP-hIPS-1-CARD fusion protein structure (pdb 2VGQ). The last helix of MBP extends and forms a continuous helix with the first helix of CARD. (**b**) Design of helical linkers. Based on the MBP-hIPS-1-CARD, one residue is inserted in the linker region incrementally from V28E1 to V28E5. The view is shown from the projection of the last helix of MBP. For clarity, only the H3 helix of the CARD is shown in cartoon and in different colors (red for V28E1, orange for V28E2, yellow for V28E3, green for V28E4 and blue for V28E5). The rotation of target protein is ~100° through the insertion of one residue in the linker. (**c**) X-ray diffraction image of zebrafish IGBP1-CARD as an MBP-fusion protein expressed with the V28E5 vector. By manipulating the linker sequences, X-ray diffraction by the crystals for the IGBP1-CARD fusion protein improved from worse than 6 Å to 1.47 Å. (**d**) Surface rendering of one MBP tagged IGBP1-CARD structure is shown. The MBP is shown in grey ribbons, the linker residues coded in the V28E5 vector in magenta and the IGBP1-CARD in green. A bound maltotetraose molecule at the MBP is shown as sticks.

To design linkers that adopt rigid structures, which can potentially reduce the conformational heterogeneity of the MBP fusion protein, we sought to model the MBP-IPS-1 CARD structure. Here the linker sequence TNSA adopts a continuous helix from the last helix of MBP to the first helix of the IPS-1 CARD which may pose minimal perturbation of the helical structures for either MBP or IPS-1 CARD^57^ (Fig. 4a). The potential drawback of such a “rigid” linker is that the relative position and orientation of the MBP and the target may be “fixed” such that the chances of packing into ordered crystals may be limited. To explore broader crystallization space, we designed a series of protein expression vectors V28E1-V28E6 containing helical linkers of different lengths (Table 1). The one residue increment of the linker length may change the orientation and location of the target proteins relative to MBP, similar to those of successive residues in an α-helix. Therefore, an increment of one residue in the linker region may allow the target protein to rotate ~100° and translate ~1.5 Å relative to MBP in solution assuming the rigidity of the linker helix (Fig. 4b). As a result, in fusion proteins expressed in vectors V28E1 to V28E6, the orientation of the target protein may change by one and a half turns along the helical axis of the last helix from MBP combined with a translation of ~9 Å. This varied spacing and orientation between MBP and targets may facilitate the presentation of different surface features of the fusion proteins and the sampling of more crystallization space (Table 1).

Using the set of V28E1-V28E6 vectors, we were able to express, purify and crystallize both the zIGBP1-CARD and the hNLRP1-PYD as MBP-fusion proteins harboring a helical linker. In contrast to crystals of poor diffraction quality using MBP-fusion protein expressed with the V28E vector, X-ray diffraction data of 1.47 Å resolutions were collected for the zIGBP1-CARD crystals using protein expressed with the V28E5 vector (Fig. 4c). This led to structural determination of the first CARD domain from the zebrafish genome^58^ (Fig. 4d). As shown in Fig. 4d, the V28E5 linker is nearly a perfect α-helix, which joins the last helix of MBP and the first helix of the CARD domain as designed. This is also one of the highest resolution structures for an MBP fusion protein, which may be attributed to the rigidity of the helical linker.

**Figure 5:**
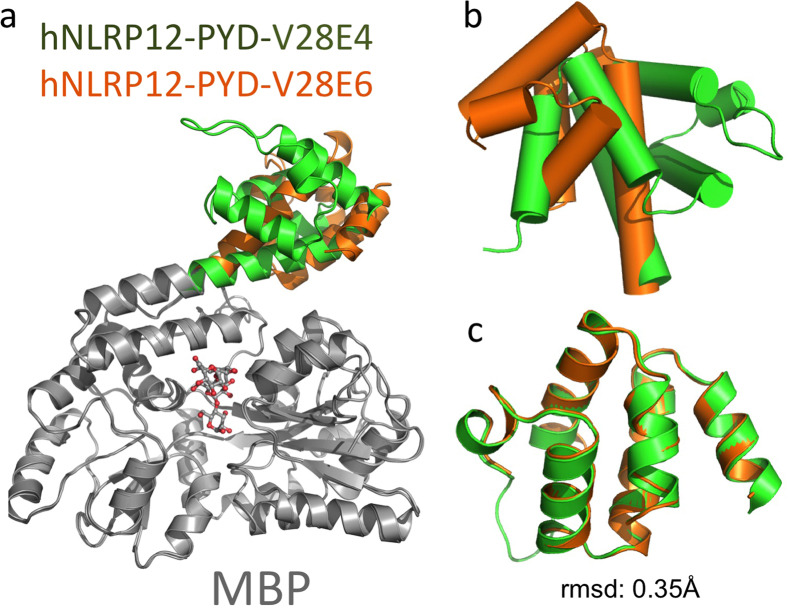
Structure of the hNLRP12-PYD crystallized in V28E4 and V28E6. (**a**) hNLRP12-PYD structures solved using fusion proteins expressed with the V28E4 and V28E6 vectors were superimposed at the MBP region. The MBP tag is colored in grey, the PYD in V28E4 is colored in green, and that in V28E6 is colored in orange. (**b**) Cylindrical presentation of hNLRP12-PYD. (**c**) Superposition of the hNLRP12-PYD structures. All of the atoms in PYD were aligned using PyMol and resulted in an rmsd of 0.35 Å.

Of note, none of the previously deposited over 100 MBP fusion protein structures were crystallized with more than one linker sequence, probably due to the challenge of the linker design and the lack of systematic approach for linker sequence selection. The set of expression vectors harboring helical linkers (V28E1-V28E6) provide researchers new opportunities to obtain crystals. Indeed, both hNLRP12-PYD and hMNDA-PYD, two targets that failed to crystallize using fusion proteins expressed with the V28E vector, crystallized using fusion proteins expressed with these new vectors (V28E1-V28E6). Remarkably, we were able to crystallize and solve their structures using MBP-fusion proteins expressed with more than one vector. In particular, the hNLRP12-PYD was crystallized as fusion proteins using both V28E4 and V28E6 vectors (Fig. 5a). In the structures, the V28E4 linker adopts a straight α-helix connecting the last helix of MBP to the first helix of PYD, whereas the V28E6 linker is slightly bent. Structural comparison reveals that the PYD rotates 220° from the V28E4 structure to the V28E6 structure (Fig. 5b). Even though the hNLRP12-PYD domains in the two crystal forms adopt different orientations relative to MBP, their structures are essentially identical with a root-mean-square deviation (rmsd) of 0.35 Å (Fig. 5b,c). This suggests that the different linker sequences and crystal lattice packing do not significantly perturb the structures of the target proteins, further validating the linker design strategy.

**Figure 6:**
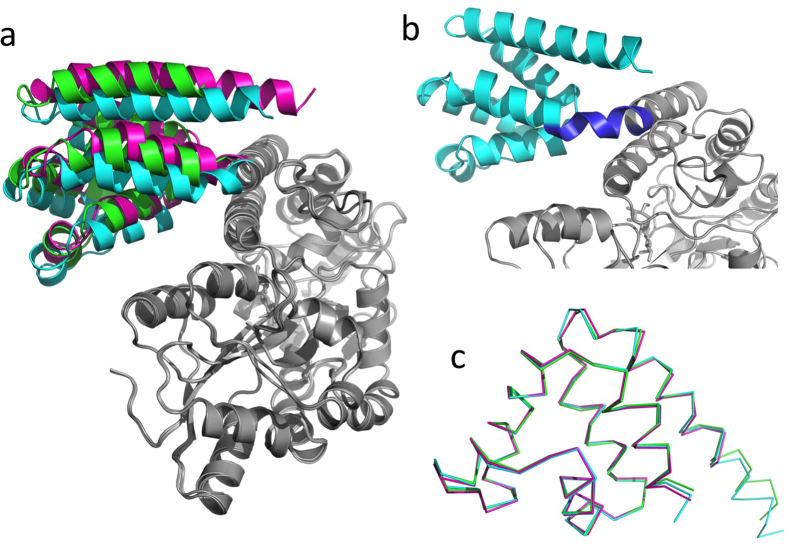
Superposition of MBP tagged hMNDA-PYD structures. (**a**) The MNDA-PYD structures crystallized in V28E3 (magenta), V28E4 (green) and V28E6 (cyan) are superimposed at the MBP region. (**b**) The V28E6 linker region (blue) forms a slightly distorted helix. (**c**) hMNDA-PYD structures solved as three fusion proteins are superimposed and are shown in ribbons. All of the atoms in PYD were aligned using PyMol and resulted in rmsds of 0.30–0.40 Å.

Similar to the hNLRP12-PYD, the human MNDA PYD domain was crystallized as MBP fusion proteins using three different protein expression vectors V28E3, V28E4, and V28E6. All of these fusion protein crystals diffract very well which led to their structure determination (Fig. 6a). Structural analysis shows that the designed “helical” linkers are slightly distorted probably attributed to crystal packing (Fig. 6b). Nonetheless, the target protein structures from all three different crystals are essentially identical with an rmsd of 0.30–0.40 Å (Fig. 6c), demonstrating again that the MBP fusion strategy enhances the crystallizability without alteration of the structural features of the target proteins.

## Conclusion and Discussion

Despite the abundance of structures for MBP fusion proteins, the design of the fusion protein expression constructs has largely been a trial-and-error process without a clear focus on the sequence or length of the linker region. There has been no report on the rational design of the linker sequences or systematic crystallization screening of MBP fusion proteins, which may have hindered the wider application of this approach in structural biology. In this study, we designed a set of seven vectors for expression of MBP-fusion proteins containing either a short flexible linker or six helical linkers. The six helical linkers were designed based on the following three considerations: first, the α helix is a relatively rigid structure which contrast with the flexible VD linker, therefore may reduce conformational heterogeneity of the fusion proteins; second, helical linkers of various lengths allows the target proteins to be positioned not only at different distances relative to the MBP protein, but also at different orientations because of the 100-degree turn and 1.5 Å translation for each successive residue at a helix. This facilitates the presentation of different surface features of the fusion proteins and the sampling of more crystallization space; third, a helical linker may facilitate the connection between the last helix of MBP and the first helix of a death domain superfamily members with minimal disruption of the helices from MBP or death domain family members. This has proven successful in determining the MBP-IPS-1-CARD structure^57^.

We tested and successfully crystallized seven targets from the challenging death domain superfamily. Two of them were crystallized in more than one crystal forms containing different linker sequences, demonstrating the power of this linker optimization strategy. We were able to determine the first crystal structures for five of them, including the hAIM2-PYD, hCARD8-CARD, zIGBP1-CARD, hNLRP12-PYD, and hMNDA-PYD. Structures of the hNLRP12-PYD and hMNDA-PYD determined with different linker sequences in different crystal lattices show no significant perturbation of the PYD structures. The structure of the hNLRP1-CARD was determined previously using a selenomethionine-labeled form at 3.1 Å resolution (3KAT). Because essentially identical structures were determined using our MBP-mediated crystallization strategy, and we observed minimal interactions between the MBP tag and the targets in our current studies, our data suggest that fusion protein tags such as MBP are unlikely to interfere with the structure or folding of death domains, and maybe other protein targets as reported previously^1^,^37^. Even though we have only tested crystallization of the death domain superfamily members, our MBP fusion strategy may promote crystallization of diverse protein targets, as was demonstrated by previously determined structures of MBP fused with targets of various sizes or folds. Nonetheless, such fusion protein strategy may not be universally applicable for certain difficult protein targets, such as intrinsically disordered proteins that may adopt different conformations depending on different fusion tags they are connected with. Therefore, the fusion protein strategy is one of many different approaches to be tested to promote crystallization.

The linker helix is relatively rigid but can still be influenced by crystal packing forces including the interactions between target protein-target protein, MBP-MBP and MBP-target protein. This may bend/distort the helix to some extent. Indeed, the bending and distortion of the linker helix is observed in some of structures in this study. For example, between the two structures of hNLRP12-PYD fusion proteins, the helix in V28E4 is a straight helix, while that in V28E6 was slightly bent. Structural comparison reveals that the PYD rotates ∼220° from the V28E4 structure to the V28E6 structure (Fig. 5a).

Highly flexible connection between the MBP tag and the target protein may be detrimental to crystallization. To best take advantage of our designed sets of vectors, we recommend removal of all flexible residues at the N-termini of the targets. Our tests demonstrated that the flexible and helical linkers may be complementary to each other in promoting crystallization. Some of our target proteins could only be crystallized with the VD linker while others only with one or more of the rigid helical linkers. Therefore, it is important to test the different linker sequences to enhance the likelihood of crystallization.

The different linker sequences may also facilitate the crystallization of protein complexes. The short linker sequence may allow the MBP tag to stabilize the target protein, but the closely positioned MBP may prevent the target protein from interacting with its partner proteins due to steric hindrance. In such cases, longer and more flexible linker sequences may be tested. The design of the helical linkers with different lengths is based on the α-helical structure that may facilitate the exposure of different surfaces of the target proteins in the context of different fusion proteins. It is therefore possible that the specific protein-protein interaction surfaces may be exposed in one or more of the designed fusion proteins.

The usage of MBP as a crystallization chaperone is perhaps partially due to its high solubility and expansive hydrophilic surface that provides versatile crystal packing interface. There are other proteins such as NusA that may possess similar properties and may be systemically tested as crystallization chaperones. It would be beneficial to test a diverse set of crystallization chaperones for a difficult protein target, because different fusion tags can provide distinct packing interfaces. One caveat of using a helical linker sequence is that the C-terminus of a different protein tag may not end with an extruded helix as in the case of MBP. In such cases, both helical and non-helical linker sequences may need to be explored.

In conclusion, our study provides a strategy for testing MBP and other crystallization chaperones through optimization of the linker sequences. We believe that the use of versatile crystallization chaperones will significantly enhance crystallizability of many challenging protein targets.

## Additional Information

**Accession codes:** Coordinates and structure factors have been deposited in the Protein Data Bank, and the accession codes are presented in Table S3.

**How to cite this article**: Jin, T. *et al*. Design of an expression system to enhance MBP-mediated crystallization. *Sci. Rep.* **7**, 40991; doi: 10.1038/srep40991 (2017).

**Publisher's note:** Springer Nature remains neutral with regard to jurisdictional claims in published maps and institutional affiliations.

## Supplementary Material

Supplement materials Table S1-S3

## Figures and Tables

**Table 1:** The MBP fusion linker sequences for the seven expression vectors.

Vector Name	Linker Sequence	Linker Structure
V28E	VD	Loop
V28E1	AAA	Helix
V28E2	AAAA	Helix
V28E3	AAAAA	Helix
V28E4	AARAAA	Helix
V28E5	AARAAAA	Helix
V28E6	AARAFAAA	Helix
