# Supplementary material for: Design of an expression system to enhance MBP-mediated crystallization
Source: Sci Rep. 2017 Jan 23;7:40991. doi: 10.1038/srep40991 (PMC5256280; doi:10.1038/srep40991)
Supplement: Supplement materials Table S1-S3 [file srep40991-s1.pdf]

Design of an expression system to enhance MBP-mediated crystallization

Tengchuan Jin[*1, 2], Watchalee Chuenchor[2], Jiansheng Jiang[2], Jinbo Cheng[1], Yajuan Li[1], Kang Fang[1], Mo Huang [2], Patrick Smith [2], and Tsan Sam Xiao[*3]

[1]Laboratory of Structural Immunology, CAS Key Laboratory of Innate Immunity and Chronic Diseases, CAS Center for Excellence in Molecular Cell Sciences, School of Life Sciences and Medical Center, University of Science and Technology of China, Hefei 230027 China

[2]Laboratory of Immunology, National Institute of Allergy and Infectious Diseases, National Institutes of Health, Bethesda, MD 20892 USA

[3]Department of Pathology, Case Western Reserve University, Cleveland, OH 44106 USA

Supplemental information includes Table S1-S3 and references.

Table S1. Crystal structures containing the MBP fusion tags.

PDB ID	Target Protein	Deposition year	PI of Target	Size of Target (residues)	Reference
**1A7L**	Dominant B-cell epitope from PRES2 region of HBV	1998	4.3	26	1
**1MG1**	HTLV-1 GP21 ectodomain	1999	7.8	84	2
**1HSJ**	SarR protein from *Staphylococcus aureus*	2000	9.3	115	3
**1MH3, 1MH4**	Yeast MATa1 homeodomain protein	2002	11	50	4
**1R6Z**	Argonaute2 PAZ domain	2003	9.2	139	5
**1NMU**	*Saccharomyces cerevisiae* ribosomal protein L30	2003	9.8	104	6
**1Y4C**	IL-4 antagonist	2004	9.4	114	7
**1T0K**	Yeast L30e-mRNA complex	2004	9.8	105	8
**1YTV**	A C-terminal fragment of the V1a vasopressin receptor	2005	9.1	66	9
**2NVU**	APPBP1-UBA3~NEDD8-NEDD8-MgATP-Ubc12(C111A), a trapped ubiquitin-like protein activation complex	2006	5.5	431	10
**2OBG**	Monobody MBP-74	2006	4.94	91	11
**2OK2**	MutS C-terminal domain	2007	4.3	35	12

**2VGQ**	Human IPS-1 CARD	2007	5.2	93	13
**2XZ3**	BLV TM hairpin	2010	8	92	14
**3A3C, 2ZXT**	TIM40/MIA40, WT and C296S, C298S mutant	2009	4.7	77	15
**3C4M**	Human parathyroid hormone in complex with the extracellular domain of its G-protein-coupled receptor (PTH1R)	2008	6.3	160	16
**3CSG, 3CSB**	Monobody YS1(MBP-74)	2008	4.5	91	17
**3D4C, 3D4G, 3EF7**	ZP-N domain of mammalian sperm receptor ZP3	2008	6.5	102	18
**3DM0**	RACK1 from A. thaliana	2008	8.1	324	19
**3EHS, 3EHT, 3EHU**	Extracellular domain of human corticotropin releasing factor receptor type 1 (CRFR1)	2008	6.3	98	20
**3F5F**	Heparan sulfate 2-O-sulfotransferase from gallus gallus	2008	8.9	288	21
**3G7V**	Islet Amyloid Polypeptide (IAPP or Amylin)	2009	8.9	37	22
**3H3G**	Extracellular domain of the human parathyroid hormone receptor (PTH1R) in complex with parathyroid hormone-related protein (PTHrP)	2009	6.3	160	23
**3H4Z**	Der p 7 protein	2009	4.9	198	24
**3HST**	N-Terminal RNASE H domain of rv2228c from *Mycobacterium tuberculosis*	2009	6.7	141	25
**3IO4, 3IO6, 3IOR, 3IOT,**	Huntingtin amino-terminal region Q17-C90	2009	10.2	79	26
**3IOU, 3IOV, 3IOW**	TRPA1 ion channel	2015	6.2	1132	27
**3L2J**	Human parathyroid hormone receptor (PTH1R)	2011	6.6	159	28
**3LBS, 3LC8**	Cytoplasmic tail of (pro)renin receptor	2010	6	19	29
**3MP1, 3MP6, 3MP8**	Complex of Sgf29 and trimethylated H3K4	2010	7.9	146	30
**3MQ9**	Ectodomain Mutant of BST-2/Tetherin/CD317	2010	5.3	86	31
**3N93, 3N95, 3N96**	Human CRFR2 alpha extracellular domain in complex with Urocortin 2	2010	4.3	102	32
**3N94**	Human pituitary adenylate cyclase 1 Receptor-short N-terminal extracellular domain	2010	4.4	97	33
**3O3U**	Human Receptor for Advanced Glycation	2010	9.8	210	34

	Endproducts (RAGE)				
**3OAI**	Extra-cellular domain of human myelin protein zero	2010	5	121	[35]
**3OB4**	Major peanut allergen Ara h 2	2010	5.8	130	[36]
**3OSR, 3OSQ**	Green fluorescent protein inserted into MBP	2010	5.9	248	[37]
**3PY7**	Full-length Bovine Papillomavirus oncoprotein E6 in complex with LD1 motif of paxillin	2010	8.2	152	[38]
**3Q25, 3Q26, 3Q27, 3Q28, 3Q29**	Human alpha-synuclein (1-19)	2011	8.3	19	[39]
**3RUM**	Glycopeptide antibiotic-target complexes	2011	N/A	5	[40]
**3VD8**	Human AIM2 PYD domain	2012	9.2	107	[41]
**3W15**	Peroxisomal targeting signal 2 (PTS2) of Saccharomyces cerevisiae 3-ketoacyl-CoA thiolase in complex with Pex7p and Pex21p	2012	8.5	17	[42]
**3WAI**	C-terminal globular domain of oligosaccharyltransferase from *Archaeoglobus fulgidus*	2013	5.2	369	[43]
**4B3N**	Rhesus TRIM5alpha PRY/SPRY domain	2012	8.6	222	[44]
**4BL8, 4BL9, 4BLB**	Full-length human Suppressor of fused (SUFU)	2013	5.1	460	N/A
**4EDQ**	Myosin-binding protein c residues 149-269	2012	6.1	121	[45]
**4DXB, 4DXC**	TEM-1 protein RG13 in complex with zigroup	2012	5.45	260	[46]
**4DEQ**	The Neuropilin-1/VEGF-A complex	2012	8.8	218	[47]
**4EGC**	Human Six1 Bound to Human Eya2 Eya Domain	2012	9.8	189	[48]
**4EXK**	The C-terminal domain of the uncharacterized protein STM14	2012	9.2	119	[49]
**4FE8, 4FEB, 4FEC, 4FED**	Htt36Q3H-EX1-X1-C1(Alpha)	2012	8.3	82	[37]
**4GIZ**	Full-length human papillomavirus oncoprotein E6 in complex with LXXLL peptide of ubiquitin ligase E6AP	2012	4.1	18	[50]
**4GLI**	Human SMN YG-Dimer	2012	9.5	37	[51]
**4H1G**	Candida albicans Kar3 motor domain	2012	8.5	344	[52]
**4IFP**	Human NLRP1-CARD domain	2012	6.5	90	[53]
**4IKM**	Human CARD8-CARD domain	2012	5	87	[54]
**4JBZ**	Mcm10 coiled-coil region	2013	6.1	32	[55]

**4KEG**	MBP Fused Human SPLUNC1	2013	5.7	220	[56]
**4KV3**	Ubiquitin-like domain of the Mycobacterium tuberculosis type VII secretion system protein EccD1	2013	4.2	114	[57]
**4KYC, 4KYD, 4KYE**	The C-terminal domain of the HPIV4B phosphoprotein	2013	10.3	56	[58]
**4LOG**	The orphan nuclear receptor PNR ligand	2013	6.7	197	[59]
**4MY2**	Cysteine-rich growth factor Norrin	2013	9.1	104	[60]
**4NUF**	SHP/EID1	2013	5.9	208	[61]
**4O2X**	A malarial protein	2013	9.3	132	[62]
**4O4B**	Inositol hexakisphosphate kinases EhlP6KA	2014	5.7	250	[63]
**4OZQ**	Mouse Kif14 motor domain	2014	6.2	349	[64]
**4PE2**	MBP PilA1 CD160	2014	4.8	138	[65]
**4PQK**	C-Terminal domain of DNA binding protein	2014	7.2	120	[66]
**4QVH**	The essential Mycobacterium tuberculosis phosphopantetheinyl transferase PptT	2014	6.2	233	[67]
**4R0Y**	C-terminal GH1 domain of Guanylate Kinase-associated Protein	2014	8	140	[68]
**4RG5**	S. Pombe SMN YG-Dimer	2014	5.9	33	[69]
**4RWF, 4RWG**	CLR:RAMP2 extracellular domain heterodimer	2014	4.8	213	[64]
**4TSM, 4OGM**	PilA1 from C. difficile R20291 residues 26-166	2014	5.8	127	[70]
**4WGI**	Myeloid Cell Leukemia 1 (MCL1)	2014	8.8	151	[71]
**4WJV**	Rsa4 in complex with the Nsa2 binding peptide	2014	4.4	22	[72]
**4WRN**	The polymerization region of human uromodulin/Tamm-Horsfall protein	2014	5.8	315	[48]
**4WTH**	Ataxin-3 Carboxy Terminal Region	2014	9.4	71	[73]
**4XAI, 4XAJ**	Red flour beetle NR2E1/TLX	2014	6	203	[74]
**4XR8**	The HPV16 E6/E6AP/p53 ternary complex	2015	4.1	18	[75]
**5AZ6, 5AZ7, 5AZ8, 5AZ9**	MBP-Tom20 fusion protein with a 2-residue spacer in the connector helix	2015	4.6	66	[75]
**5AZA**	MBP-sAglB fusion protein with a 20-residue spacer in the connector helix	2015	5.3	492	[76]
**5C7R**	An antifreeze protein	2015	4.9	73	[77]
**5CBN**	B4 domain of protein A from	2015	4.3	176	[78]

	staphylococcal aureus with chemical cross-linker EY-CBS				
**5CL1**	Norris with human Frizzled 4	2015	6.2	131	[79]
**5DFM**	Tetrahymena telomerase p19	2015	8	170	[80]
**5E24**	Su(H)-Hairless-DNA Repressor Complex	2015	5.1	370	[81]
**5EDV**	The HOIP-RBR/UbcH5B	2015	7	380	[82]
**5HZ7**	Minor DNA-binding pilin ComP from Neisseria meningitidis	2016	9.3	125	[83]
**5I69**	MamC magnetite-interaction component mutant-D70A	2016	8.5	28	[84]
**5IQZ**	N-terminal domain of Human SIRT7	2016	11.5	78	[85]

Table S2. Protein sequences tested for crystallization.

Target	Sequences[1,2,3]
hNLRP1-CARD	LHFVDQYREQLIARVTSVEVVLDKLHGQVLSQEQYERVLAENTR PSQMRKLFSLSQSWDRKCKDGLYQALKETHPHLIMELWE
hAIM2-PYD	MESKYKEILLLTGLDNITDEELDRFKAALSDEFNIATGKLHTANRI QVATLMIQNAGAVSAVMKTIRIFQKLNYMLLAKRLQEEKEKVD KQYKSVTKPKPLSQAEMS
hCARD8-CARD	AAFVKENHRQLQARMGDLKGVLDDLQDNEVLTENEKELVEQEK TRQSKNEALLSMVEKKGDLALDVLFRSISERDPYLVSYLRQQNL
zGBP1-CARD	SEFVDALRGDLIQKVSSVMAIADSLMSERMITDELYNEVHNADT NQRKMRLLFRALDSGGASVKAEFYRLLMENEPRLVHELESRHSE SSGPQ
hNLRP1-PYD1	AWGRLACYLEFLKKEELKEFQLLLANKAHSRSSSGETPAQPEKTS GMEVASYLVAQYGEQRAWDLALHTWEQMGLRSLCAQAQE
[4]hNLRP1-PYD2	RLACYLEFLKKEELKEFQLLLANKAHSRSSSGETPAQPEKTSGME VASYLVAQYGEQRAWDLALHTWEQMGLRSLCAQAQE
hNLRP12-PYD	LCRLSTYLEELEAVELKKFKLYLGTATELGEGKIPWGSMEKAGPL EMAQLLITHFGPEEAWRLALSTFERINRKDLWERGQREDLVRDT PPGGPSS
hMNDA-PYD	EYKKILLLKGFELMDDYHFTSIKSLLAYDLGLTTKMQEEYNRIKI TDLMEKKFQGVACLDKLIELAKDMPSLKNLVNNLRKEKSKVAK KIKTQEK
[5]MBP	MKIEEGKLVIWINGDKGYNGLAEVGKKFEKDTGIKVTVEHPDKL EEKFPQVAATGDGPDIIFWAHDRFGGYAQSGLLAEITPAAAFQDK LYPFTWDAVRYNGKLIAYPIAVEALSLIYNKDLLPNPPKTWEEIP ALDKELKAKGKSALMFNLQEPYFTWPLIAADGGYAFKYAAGKY DIKDVGVDNAGAKAGLTFLVDLIKNKHMNADTDYSIAEAAFNK GETAMTINGPWAWSNIDTSAVNYGVTVLPTFKGQPSKPFVGVLS AGINAASPNKELAKEFLENYLLTDEGLEAVNKDKPLGAVALKSY EEELAKDPRIAATMENAQKGEIMPNIPQMSAFWYAVRTAVINAA SGRQTVDAALAAAQTNA

Note:

1. The same 8 sequences from 7 different death fold proteins were ligated into a cleavable MBP tagged vector, and 7 non-cleavable MBP tagged vectors, ie V28E and V28E1-E6 for protein expression and crystallization screening. A total of 64 constructs were tested.
2. For the non-tagged crystallization trials, the final protein sequence after MBP tag removal has a four amino acids (GSVD) addition at its N-terminus, and a four amino acids (AAAS) addition at its C-terminus due to cloning sites.
3. For MBP-tag aided crystallization trials, the final protein sequence is designed to have the mutant MBP plus linker (as shown in Table 1) at the N-termini, and LEHHHHH at the C-termini.
4. Two different boundaries for hNLRP1-PYD were tested as we failed to solve its crystal structure with the method described in this manuscript.
5. A mutant form of MBP is used in all of our co-crystallization vectors. By mutating five hydrophilic residues, this mutant form of MBP is reported to be superior in crystallization[86].

Table S3. X-ray data collection and refinement table.

	hNLRP12-PYD-V28E4	hNLRP12-PYD-V28E6	hMNDA-PYD-V28E3	hMNDA-PYD-V28E4	hMNDA-PYD-V28E6
Crystallization condition	30% PEG550, 0.2M Ammonium Sulfate, NaAc 4.6	3.5M NaFormate, NaAc 4.6	15% PEG8000, 0.2M NH4Ac, NaAc 5.0	15% PEG4000, 0.2 M NH4Ac, 0.1 M NaAc 5.0	20% PEG4000, 0.1 M Tris-HCl 8.0
Cryo-protectant	30% PEG550, 20% Ethylene Glycol, 0.2M Ammonium Sulfate, NaAc 4.6	3.5M NaFormate, 20% Glucose, NaAc 4.6	15% PEG8000, 20% Ethylene Glycol, 10% Glycerol, 5% MAL, 0.2M NH4Ac, NaAc 5.0	15% PEG4000, 20% Ethylene Glycol, 0.2 M NH4Ac, 0.1 M NaAc 5.0	20% PEG4000, 20% Ethylene Glycol, 0.1 M Tris-HCl 8.0
Data set name	TJ100-6	TJ97-3	TJ121-1	TJ104-2	TJ108-12
Space group	$P2_1$	$P2_12_12$	$P2_1$	$P2_12_12$	$P2_12_12_1$
Unit cell (a, b, c) (Å)	42.5, 104.5, 110.9	103.6, 186.7, 52.7	73.0, 186.2, 76.4	192.2， 235.6， 73.7	42.0, 68.8, 174.4
(α,β,γ) (°)	90.0, 98.9, 90.0	90, 90, 90	90, 89.8, 90	90, 90, 90	90, 90, 90
Resolution (Å) *	50-1.85 (1.90-1.85)	50 - 1.70 (1.80-1.70)	50-1.65 (1.71-1.65)	50-2.00 (2.12-2.00)	50-1.45 (1.54-1.45)
No. of reflections (total/unique)	461011/80635	670878/110662	895581/241934	2753578/201859	529651/89636
Redundancy*	5.7 (5.6)	6.1 (3.1) *	3.7 (3.6)	13.6 (12.4)	5.9 (4.1)
Completeness (%)*	98.6 (96.0)	97.5 (85.3) *	99.0 (95.3)	89.0 (53.4)	98.9 (95.7)
I/σ(I) *	17.3 (1.7)	22.4 (3.7) *	11.7 (1.8)	20.4 (3.4)	23.1 (2.1)
R-meas ¶*	0.060 (1.357)	0.055 (0.323)	0.072 (0.864)	0.098 (0.741)	0.039 (0.730)
CC(1/2) *	0.998 (0.636)	0.999 (0.892)	0.998 (0.708)	0.999 (0.937)	0.999 (0.800)
Refinement					
Resolution (Å)	50-1.85	50-1.70	50-1.65	50-2.00	50-1.45
No. of protein atoms	7141	7175	14620	21517	3666
No. of solvent/hetero-atoms	399/147	1081/65	1949/125	1142/138	470/32
Rmsd bond lengths (Å)	0.014	0.007	0.006	0.003	0.006
Rmsd bond angles (°)	1.31	1.00	0.80	0.57	0.79
Rwork†	0.18	0. 1604	0.1783	0.221	0.207

Rfree‡	0.224	0.2009	0.2123	0.269	0.225
Ramachandran plot (favored/disallowed) **	97.9/0	98.8/0	99.2/0	97.9/0	98.9/0
PDB code	5H7N	4XHS	5WQ6	5WPZ	5H7Q

*Asterisked numbers correspond to the last resolution shell.

¶ $R_{meas} = \Sigma_h(n/n-1)^{1/2} \Sigma_i |I_i(h) - \langle I(h) \rangle| / \Sigma_h\Sigma_i I_i(h)$, where $I_i(h)$ and $\langle I(h) \rangle$ are the ith and mean measurement of the intensity of reflection *h*.

† $R_{work} = \Sigma_h||F_{obs}(h)|-|F_{calc}(h)|| / \Sigma_h|F_{obs}(h)|$, where $F_{obs}(h)$ and $F_{calc}(h)$ are the observed and calculated structure factors, respectively. No I/σ cutoff was applied.

‡R_{free} is the R value obtained for a test set of reflections consisting of a randomly selected 10% subset of the data set excluded from refinement.

**Values from Molprobity server (http://molprobity.biochem.duke.edu/).

References:

1. Saul, F.A., Vulliez-le Normand, B., Lema, F. & Bentley, G.A. Crystal structure of a dominant B-cell epitope from the preS2 region of hepatitis B virus in the form of an inserted peptide segment in maltodextrin-binding protein. *J Mol Biol* **280**, 185-92 (1998).
2. Kobe, B., Center, R.J., Kemp, B.E. & Poumbourios, P. Crystal structure of human T cell leukemia virus type 1 gp21 ectodomain crystallized as a maltose-binding protein chimera reveals structural evolution of retroviral transmembrane proteins. *Proc Natl Acad Sci U S A* **96**, 4319-24 (1999).
3. Liu, Y. et al. Crystal structure of the SarR protein from Staphylococcus aureus. *Proc Natl Acad Sci U S A* **98**, 6877-82 (2001).
4. Ke, A. & Wolberger, C. Insights into binding cooperativity of MATa1/MATalpha2 from the crystal structure of a MATa1 homeodomain-maltose binding protein chimera. *Protein Sci* **12**, 306-12 (2003).

5. Song, J.J. et al. The crystal structure of the Argonaute2 PAZ domain reveals an RNA binding motif in RNAi effector complexes. *Nat Struct Biol* **10**, 1026-32 (2003).
6. Chao, J.A., Prasad, G.S., White, S.A., Stout, C.D. & Williamson, J.R. Inherent protein structural flexibility at the RNA-binding interface of L30e. *J Mol Biol* **326**, 999-1004 (2003).
7. Laporte, S.L. et al. De novo design of an IL-4 antagonist and its structure at 1.9 A. *Proc Natl Acad Sci U S A* **102**, 1889-94 (2005).
8. Chao, J.A. & Williamson, J.R. Joint X-ray and NMR refinement of the yeast L30e-mRNA complex. *Structure* **12**, 1165-76 (2004).
9. Adikesavan, N.V. et al. A C-terminal segment of the V1R vasopressin receptor is unstructured in the crystal structure of its chimera with the maltose-binding protein. *Acta Crystallogr Sect F Struct Biol Cryst Commun* **61**, 341-5 (2005).
10. Huang, D.T. et al. Basis for a ubiquitin-like protein thioester switch toggling E1-E2 affinity. *Nature* **445**, 394-8 (2007).
11. Koide, A., Gilbreth, R.N., Esaki, K., Tereshko, V. & Koide, S. High-affinity single-domain binding proteins with a binary-code interface. *Proc Natl Acad Sci U S A* **104**, 6632-7 (2007).
12. Mendillo, M.L., Putnam, C.D. & Kolodner, R.D. Escherichia coli MutS tetramerization domain structure reveals that stable dimers but not tetramers are essential for DNA mismatch repair in vivo. *J Biol Chem* **282**, 16345-54 (2007).
13. Potter, J.A., Randall, R.E. & Taylor, G.L. Crystal structure of human IPS-1/MAVS/VISA/Cardif caspase activation recruitment domain. *BMC Struct Biol* **8**, 11 (2008).
14. Lamb, D., Schuttelkopf, A.W., van Aalten, D.M. & Brighty, D.W. Charge-surrounded pockets and electrostatic interactions with small ions modulate the activity of retroviral fusion proteins. *PLoS Pathog* **7**, e1001268 (2011).
15. Kawano, S. et al. Structural basis of yeast Tim40/Mia40 as an oxidative translocator in the mitochondrial intermembrane space. *Proc Natl Acad Sci U S A* **106**, 14403-7 (2009).

16. Pioszak, A.A. & Xu, H.E. Molecular recognition of parathyroid hormone by its G protein-coupled receptor. *Proc Natl Acad Sci U S A* **105**, 5034-9 (2008).
17. Gilbreth, R.N., Esaki, K., Koide, A., Sidhu, S.S. & Koide, S. A dominant conformational role for amino acid diversity in minimalist protein-protein interfaces. *J Mol Biol* **381**, 407-18 (2008).
18. Monne, M., Han, L., Schwend, T., Burendahl, S. & Jovine, L. Crystal structure of the ZP-N domain of ZP3 reveals the core fold of animal egg coats. *Nature* **456**, 653-7 (2008).
19. Ullah, H. et al. Structure of a signal transduction regulator, RACK1, from Arabidopsis thaliana. *Protein Sci* **17**, 1771-80 (2008).
20. Pioszak, A.A., Parker, N.R., Suino-Powell, K. & Xu, H.E. Molecular recognition of corticotropin-releasing factor by its G-protein-coupled receptor CRFR1. *J Biol Chem* **283**, 32900-12 (2008).
21. Bethea, H.N., Xu, D., Liu, J. & Pedersen, L.C. Redirecting the substrate specificity of heparan sulfate 2-O-sulfotransferase by structurally guided mutagenesis. *Proc Natl Acad Sci U S A* **105**, 18724-9 (2008).
22. Wiltzius, J.J., Sievers, S.A., Sawaya, M.R. & Eisenberg, D. Atomic structures of IAPP (amylin) fusions suggest a mechanism for fibrillation and the role of insulin in the process. *Protein Sci* **18**, 1521-30 (2009).
23. Pioszak, A.A., Parker, N.R., Gardella, T.J. & Xu, H.E. Structural basis for parathyroid hormone-related protein binding to the parathyroid hormone receptor and design of conformation-selective peptides. *J Biol Chem* **284**, 28382-91 (2009).
24. Mueller, G.A. et al. The structure of the dust mite allergen Der p 7 reveals similarities to innate immune proteins. *J Allergy Clin Immunol* **125**, 909-917 e4 (2010).
25. Watkins, H.A. & Baker, E.N. Structural and functional characterization of an RNase HI domain from the bifunctional protein Rv2228c from Mycobacterium tuberculosis. *J Bacteriol* **192**, 2878-86 (2010).
26. Kim, M.W., Chelliah, Y., Kim, S.W., Otwinowski, Z. & Bezprozvanny, I. Secondary structure of Huntingtin amino-terminal region. *Structure* **17**, 1205-12 (2009).
27. Paulsen, C.E., Armache, J.P., Gao, Y., Cheng, Y. & Julius, D. Structure of the TRPA1 ion channel suggests regulatory mechanisms. *Nature* (2015).

28. Zhang, Y., Gao, X. & Michael Garavito, R. Structural analysis of the intracellular domain of (pro)renin receptor fused to maltose-binding protein. *Biochem Biophys Res Commun* **407**, 674-9 (2011).
29. Bian, C. et al. Sgf29 binds histone H3K4me2/3 and is required for SAGA complex recruitment and histone H3 acetylation. *EMBO J* **30**, 2829-42 (2011).
30. Yang, H. et al. Structural insight into the mechanisms of enveloped virus tethering by tetherin. *Proc Natl Acad Sci U S A* **107**, 18428-32 (2010).
31. Pal, K., Swaminathan, K., Xu, H.E. & Pioszak, A.A. Structural basis for hormone recognition by the Human CRFR2{alpha} G protein-coupled receptor. *J Biol Chem* **285**, 40351-61 (2010).
32. Kumar, S., Pioszak, A., Zhang, C., Swaminathan, K. & Xu, H.E. Crystal structure of the PAC1R extracellular domain unifies a consensus fold for hormone recognition by class B G-protein coupled receptors. *PLoS One* **6**, e19682 (2011).
33. Park, H., Adsit, F.G. & Boyington, J.C. The 1.5 A crystal structure of human receptor for advanced glycation endproducts (RAGE) ectodomains reveals unique features determining ligand binding. *J Biol Chem* **285**, 40762-70 (2010).
34. Liu, Z. et al. Crystal structure of the extracellular domain of human myelin protein zero. *Proteins* **80**, 307-13 (2012).
35. Mueller, G.A. et al. Ara h 2: crystal structure and IgE binding distinguish two subpopulations of peanut allergic patients by epitope diversity. *Allergy* **66**, 878-85 (2011).
36. Marvin, J.S., Schreiter, E.R., Echevarria, I.M. & Looger, L.L. A genetically encoded, high-signal-to-noise maltose sensor. *Proteins* **79**, 3025-36 (2011).
37. Zanier, K. et al. Structural basis for hijacking of cellular LxxLL motifs by papillomavirus E6 oncoproteins. *Science* **339**, 694-8 (2013).
38. Zhao, M., Cascio, D., Sawaya, M.R. & Eisenberg, D. Structures of segments of alpha-synuclein fused to maltose-binding protein suggest intermediate states during amyloid formation. *Protein Sci* **20**, 996-1004 (2011).
39. Economou, N.J. et al. A carrier protein strategy yields the structure of dalbavancin. *J Am Chem Soc* **134**, 4637-45 (2012).
40. Jin, T., Curry, J., Smith, P., Jiang, J. & Xiao, T.S. Structure of the NLRP1 caspase recruitment domain suggests potential mechanisms for its association with procaspase-1. *Proteins* **81**, 1266-70 (2013).

41. Pan, D., Nakatsu, T. & Kato, H. Crystal structure of peroxisomal targeting signal-2 bound to its receptor complex Pex7p-Pex21p. *Nat Struct Mol Biol* **20**, 987-93 (2013).
42. Matsumoto, S., Shimada, A. & Kohda, D. Crystal structure of the C-terminal globular domain of the third paralog of the Archaeoglobus fulgidus oligosaccharyltransferases. *BMC Struct Biol* **13**, 11 (2013).
43. Yang, H. et al. Structural insight into HIV-1 capsid recognition by rhesus TRIM5alpha. *Proc Natl Acad Sci U S A* **109**, 18372-7 (2012).
44. Cherry, A.L. et al. Structural basis of SUFU-GLI interaction in human Hedgehog signalling regulation. *Acta Crystallogr D Biol Crystallogr* **69**, 2563-79 (2013).
45. Ke, W. et al. Structure of an engineered beta-lactamase maltose binding protein fusion protein: insights into heterotropic allosteric regulation. *PLoS One* **7**, e39168 (2012).
46. Parker, M.W., Xu, P., Li, X. & Vander Kooi, C.W. Structural basis for selective vascular endothelial growth factor-A (VEGF-A) binding to neuropilin-1. *J Biol Chem* **287**, 11082-9 (2012).
47. Patrick, A.N. et al. Structure-function analyses of the human SIX1-EYA2 complex reveal insights into metastasis and BOR syndrome. *Nat Struct Mol Biol* **20**, 447-53 (2013).
48. Zhemkov, V.A., Kulminskaya, A.A., Bezprozvanny, I.B. & Kim, M. The 2.2-Angstrom resolution crystal structure of the carboxy-terminal region of ataxin-3. *FEBS Open Bio* **6**, 168-78 (2016).
49. Kim, M. Beta conformation of polyglutamine track revealed by a crystal structure of Huntingtin N-terminal region with insertion of three histidine residues. *Prion* **7**, 221-8 (2013).
50. Martin, R., Gupta, K., Ninan, N.S., Perry, K. & Van Duyne, G.D. The survival motor neuron protein forms soluble glycine zipper oligomers. *Structure* **20**, 1929-39 (2012).
51. Delorme, C., Joshi, M. & Allingham, J.S. Crystal structure of the Candida albicans Kar3 kinesin motor domain fused to maltose-binding protein. *Biochem Biophys Res Commun* **428**, 427-32 (2012).
52. Jin, T., Huang, M., Smith, P., Jiang, J. & Xiao, T.S. The structure of the CARD8 caspase-recruitment domain suggests its association with the FIIND domain and

procaspases through adjacent surfaces. *Acta Crystallogr Sect F Struct Biol Cryst Commun* **69**, 482-7 (2013).

53. Jin, T., Huang, M., Smith, P., Jiang, J. & Xiao, T.S. Structure of the caspase-recruitment domain from a zebrafish guanylate-binding protein. *Acta Crystallogr Sect F Struct Biol Cryst Commun* **69**, 855-60 (2013).
54. Du, W. et al. Mcm10 self-association is mediated by an N-terminal coiled-coil domain. *PLoS One* **8**, e70518 (2013).
55. Ning, F. et al. Structural characterization of the pulmonary innate immune protein SPLUNC1 and identification of lipid ligands. *FASEB J* **28**, 5349-60 (2014).
56. Wagner, J.M. et al. Structures of EccB1 and EccD1 from the core complex of the mycobacterial ESX-1 type VII secretion system. *BMC Struct Biol* **16**, 5 (2016).
57. Yegambaram, K., Bulloch, E.M. & Kingston, R.L. Protein domain definition should allow for conditional disorder. *Protein Sci* **22**, 1502-18 (2013).
58. Tan, M.H. et al. The crystal structure of the orphan nuclear receptor NR2E3/PNR ligand binding domain reveals a dimeric auto-repressed conformation. *PLoS One* **8**, e74359 (2013).
59. Ke, J. et al. Structure and function of Norrin in assembly and activation of a Frizzled 4-Lrp5/6 complex. *Genes Dev* **27**, 2305-19 (2013).
60. Zhi, X. et al. Structural insights into gene repression by the orphan nuclear receptor SHP. *Proc Natl Acad Sci U S A* **111**, 839-44 (2014).
61. AhYoung, A.P., Koehl, A., Vizcarra, C.L., Cascio, D. & Egea, P.F. Structure of a putative ClpS N-end rule adaptor protein from the malaria pathogen Plasmodium falciparum. *Protein Sci* **25**, 689-701 (2016).
62. Wang, H., DeRose, E.F., London, R.E. & Shears, S.B. IP6K structure and the molecular determinants of catalytic specificity in an inositol phosphate kinase family. *Nat Commun* **5**, 4178 (2014).
63. Arora, K. et al. KIF14 binds tightly to microtubules and adopts a rigor-like conformation. *J Mol Biol* **426**, 2997-3015 (2014).
64. Piepenbrink, K.H. et al. Structural and evolutionary analyses show unique stabilization strategies in the type IV pili of Clostridium difficile. *Structure* **23**, 385-96 (2015).

65. Schumacher, M.A. et al. Mechanism of staphylococcal multiresistance plasmid replication origin assembly by the RepA protein. *Proc Natl Acad Sci U S A* **111**, 9121-6 (2014).
66. Jung, J. et al. Crystal structure of the essential Mycobacterium tuberculosis phosphopantetheinyl transferase PptT, solved as a fusion protein with maltose binding protein. *J Struct Biol* **188**, 274-278 (2014).
67. Tong, J., Yang, H., Eom, S.H., Chun, C. & Im, Y.J. Structure of the GH1 domain of guanylate kinase-associated protein from Rattus norvegicus. *Biochem Biophys Res Commun* **452**, 130-5 (2014).
68. Gupta, K. et al. Oligomeric Properties of Survival Motor Neuron.Gemin2 Complexes. *J Biol Chem* (2015).
69. Booe, J.M. et al. Structural Basis for Receptor Activity-Modifying Protein-Dependent Selective Peptide Recognition by a G Protein-Coupled Receptor. *Mol Cell* (2015).
70. Fang, C. et al. Single Diastereomer of a Macrolactam Core Binds Specifically to Myeloid Cell Leukemia 1 (MCL1). *ACS Med Chem Lett* **5**, 1308-12 (2014).
71. Bassler, J. et al. A network of assembly factors is involved in remodeling rRNA elements during preribosome maturation. *J Cell Biol* **207**, 481-98 (2014).
72. Demuynck, S., Succiu, I.R., Grumiaux, F., Douay, F. & Lepretre, A. Effects of field metal-contaminated soils submitted to phytostabilisation and fly ash-aided phytostabilisation on the avoidance behaviour of the earthworm Eisenia fetida. *Ecotoxicol Environ Saf* **107**, 170-7 (2014).
73. Zhi, X. et al. Structural basis for corepressor assembly by the orphan nuclear receptor TLX. *Genes Dev* **29**, 440-50 (2015).
74. Martinez-Zapien, D. et al. Structure of the E6/E6AP/p53 complex required for HPV-mediated degradation of p53. *Nature* **529**, 541-5 (2016).
75. Matsuoka, R., Shimada, A., Komuro, Y., Sugita, Y. & Kohda, D. Rational design of crystal contact-free space in protein crystals for analyzing spatial distribution of motions within protein molecules. *Protein Sci* (2015).
76. Sun, T., Gauthier, S.Y., Campbell, R.L. & Davies, P.L. Revealing Surface Waters on an Antifreeze Protein by Fusion Protein Crystallography. *J Phys Chem B* (2015).
77. Jeong, W.H. et al. Connecting two proteins using a fusion alpha helix stabilized by a chemical cross linker. *Nat Commun* **7**, 11031 (2016).

78. Shen, G. et al. Structural basis of the Norrin-Frizzled 4 interaction. *Cell Res* (2015).
79. Jiang, J. et al. Structure of Tetrahymena telomerase reveals previously unknown subunits, functions, and interactions. *Science* **350**, aab4070 (2015).
80. Yuan, Z. et al. Structure and Function of the Su(H)-Hairless Repressor Complex, the Major Antagonist of Notch Signaling in Drosophila melanogaster. *PLoS Biol* **14**, e1002509 (2016).
81. Lechtenberg, B.C. et al. Structure of a HOIP/E2~ubiquitin complex reveals RBR E3 ligase mechanism and regulation. *Nature* **529**, 546-50 (2016).
82. Berry, J.L. et al. A Comparative Structure/Function Analysis of Two Type IV Pilin DNA Receptors Defines a Novel Mode of DNA Binding. *Structure* (2016).
83. Nudelman, H. et al. Structure-function studies of the magnetite-biomineralizing magnetosome-associated protein MamC. *J Struct Biol* (2016).
84. Priyanka, A., Solanki, V., Parkesh, R. & Thakur, K.G. Crystal structure of the N-terminal domain of human SIRT7 reveals a three-helical domain architecture. *Proteins* (2016).
85. Pham, P. et al. Structural analysis of the activation-induced deoxycytidine deaminase required in immunoglobulin diversification. *DNA Repair (Amst)* **43**, 48-56 (2016).
86. Moon, A.F., Mueller, G.A., Zhong, X. & Pedersen, L.C. A synergistic approach to protein crystallization: combination of a fixed-arm carrier with surface entropy reduction. *Protein Sci* **19**, 901-13 (2010).
